# Occipito-Posterior Positions1Read before the Central New York Medical Association, Buffalo, N. Y., October 16, 1894.

**Published:** 1895-03

**Authors:** P. W. Van Peyma

**Affiliations:** Buffalo, N. Y., Obstetrician to the Buffalo General and Erie County Hospitals; 445 William Street


### OCCIPITO-POSTERIOR POSITIONS.[1]

BY P. W. VAN PEYMA, M. D., Buffalo, N. Y.,

Obstetrician to the Buffalo General and Erie County Hospitals.

MY OWN experience and that of several of my colleagues, as well as the varying and often contradictory views expressed by writers on obstetrics, lead me to the conviction that the treatment of occipito-posterior positions is a subject which merits further attention.

The comparative frequency of the occurrence of this variety of vertex presentations adds to the importance of this subject. While different statistics naturally vary somewhat, it is generally admitted that fully one-fourth originally present with the occiput posteriorly to a greater or less degree.

Unscientific obstetrics, as practised in these cases, leads to many deplorable results. Penrose says: "If I were to be asked what one obstetric difficulty, in my experience, had caused most maternal and fetal deaths, what one had caused most maternal and fetal accidents, not necessarily fatal,—accidents, however, often making the rest of life worthless, or still worse than worthless, a tragedy,—I think I would say occipito-posterior positions, where the occiput had rotated into the hollow of the sacrum, and which had been *improperly treated.*" My own observation warrants similar conclusions.

Proper treatment presupposes and demands correct diagnosis. The early diagnosis of the condition is not only of vital importance, but is also often difficult. The determination of the various positions of the vertex, by a recognition of the fontanelles and sutures, implies a practical knowledge not easily acquired. As a consequence the average physician continues to apply the forceps

---

1. Read before the Central New York Medical Association, Buffalo, N. Y., October 16, 1894.

in protracted cases of this kind, regardless of the position, and as though the head were a perfect sphere and the pelvis a perfect circle. Naturally we find that, in numerous instances, the head is drawn through with the occiput posteriorly, where, if the case had received intelligent treatment, or even had been left to nature, anterior rotation would have been obtained, with a more favorable result for both mother and child.

I desire here to call attention to the triangular depression presented by the occipital bone, when, as a result of intrapelvic pressure, it is overlapped by the edges of the parietal bones, as being, perhaps, the most characteristic landmark presented by the vertex. In consequence of the varying relation of the body to the head, as well as on account of the inherent difficulties of the method, external abdominal examination is often unsatisfactory. In rare cases it may be necessary to reach an ear, or even the face, in order to arrive at a positive diagnosis.

Regarding the nature of these cases, there can scarcely be any doubt that the large majority should be considered as normal presentations, in which nature is entirely adequate to effect a normal delivery. A very great proportion of these eventually result in forward rotation of the occiput. According to Kehrer, quoted by Schröder, only one vertex in seventy-five is delivered with the occiput posteriorly. Considering this fact, in connection with the fact already referred to, that at least one in four vertex presentations originally presents with the occiput posteriorly, we see that only about one in twenty fails to rotate normally. American statistics give rather less favorable results. This I am inclined to attribute to the greater tendency in this country to interfere—to the earlier and more frequent employment of the forceps.

Forward rotation, or its failure, depends upon many circumstances. With pronounced flexion, strong pains, a normal perineum and the head and pelvis of normal size and shape, we can confidently look forward to natural rotation. In proportion as these conditions are lacking, will the frequency of failure increase. Many cases occurring in my own practice might be cited to illustrate these statements, which are also in accordance with the views expressed by many writers. And yet, while it is maintained that the majority of these cases run a normal course, it cannot be denied that, as a rule, they are unusually protracted and also not rarely call for intelligent intervention. The less easy adaptation of the larger occipital end to the comparatively smaller posterior lateral portion of the inlet, the greater rotation required and the reversed leverage through which the *vis a tergo* is applied, are the more important factors which account for the frequent delay, trying to both patient and accoucheur.

The treatment which may be required varies, naturally, with the stage of labor. The general rule, to maintain the membranes intact during the first stage, applies here with unusual force. Postural treatment, recommended by Reynolds and others, in the expectation of effecting rotation by the force of gravity, has not, in my experience, been very successful. Objections to it are that, if long continued, it causes exhaustion of the patient, and that by calling her attention to the irregular condition it also affects unfavorably her morale. The mental state of the patient is a factor of great importance and one quite frequently underrated or entirely ignored. In arriving at a conclusion as to the necessity for active interference, many circumstances require consideration. Assuming that the circumstances are otherwise normal, we shall do well to leave the case to nature, so long as fetal heart-sounds are strong and regular and we have the confidence and coöperation of the mother, the latter based on a good physical and mental state. If, however, the case becomes unduly prolonged and the condition of fetus, or mother, or both, demand interference, it will often be found that the indication is fulfilled by effecting and maintaining more pronounced flexion. The importance of complete flexion depends upon the well-known fact that the lowest point, in this case the occiput, is naturally deflected forward. Flexion may sometimes be brought about by upward pressure, especially during a pain, with the tips of one or more fingers upon the forehead. This procedure may sometimes be advantageously supplemented by external pressure upon the body of the fetus with the other hand. According to my experience this method is less positive in its results, especially when the head is still but little engaged, than it has been made to appear by many writers on obstetrics. On the other hand I have been much impressed with the mobility of the head, even when well advanced into the excavation, if the patient be thoroughly anesthetized. If the former procedure fails to accomplish its object, the patient may be anesthetized and the rectification affected by means of the whole hand. The use of the vectis, or of one blade of the forceps, I have found useful in certain cases. If, by means of the finger tips, flexion is not accomplished ; or, if having been obtained, partial extension reoccurs ; or, if with complete flexion there is no advance, then more active interference is proper—is, in fact, demanded.

Under these circumstances the patient should be completely anesthetized, the whole hand introduced and partial rotation of the occiput effected, following which the forceps may be applied advantageously, less for the purpose of immediate traction than to maintain the position of the head. It may even be a question whether this is not the proper treatment when it has become necessary to introduce the whole hand, rather than to limit the operation, as has just been explained, in certain cases to the production of flexion.

Podalic version is an alternative which naturally presents itself for consideration. I am satisfied that, except in cases demanding rapid extraction, version by the feet cannot compete with the procedure described. In addition to the conditions commonly enumerated as more or less contra-indicating podalic version and making it dangerous for either mother or child, primiparity should also be considered, especially in its relation to fetal mortality, by its interference with rapid extraction of the head.

In the application of the forceps, following partial rotation, it may frequently be well to rest satisfied with an approximately correct relation of the blades to the head, to be followed by a more perfect reapplication after the head has descended and rotation also, perhaps, farther advanced.

The application of the forceps without previous rotation and with tips to forehead, in the expectation that the occiput will rotate posteriorly, should be avoided wherever possible and limited to those cases where the head is so deeply and firmly engaged that rectification is impossible or dangerous.

The application of the reversed forceps to the occiput, thereby producing flexion, I have never tried. The procedure is one, probably, rarely to be preferred.

Where the circumstances demand the application of the forceps, with the occiput posteriorly, the blades should be applied rather farther back than in occiput anterior positions, the object being here, as in face presentation with the chin anteriorly, to obtain a larger and firmer hold. The handles will, therefore, be less depressed in these cases. Tractions, under these circumstances, should be backward according to the axes of the succeeding planes, until the head reaches the perineum, then sharply forward, using the symphysis as a fulcrum, maintaining extreme flexion until the perineum slips over the occiput, when the handles are suddenly dropped and the forceps removed.

This condition is the analogue of face presentations with the chin backward. In both, the presenting part must travel a long distance before the mass engaged can be resolved—in the one instance by extension, in the other by flexion—into its component factors, the head and trunk. Necessarily the dangers to both mother and child are greatly increased.

In conclusion, I desire to emphasize the vital importance of recognizing the position in vertex presentations. To insist that, as a rule, cases of occipito-posterior position should be left for the natural forces to effect delivery—forces which, in the vast majority of cases, are not only entirely adequate, but in these cases will accomplish the object better than the most skilled instrumental or manual interference.

Further, I desire to maintain that flexion is essential to natural rotation; that rotation is frequently delayed until the head is very low; that the character of the pains is a very important factor; that with complete anesthesia the mobility of the head, even when deep in the excavation, is often quite surprising; that in occipito-posterior position the blades of the forceps must be applied well forward to insure a firm hold; and that, after the head reaches the perineum, extreme flexion must be maintained until the occiput has passed over the perineum; and, lastly, that no hard and fast rules can be formulated to cover all cases, but that much must neces sarily be left to the judgment of the operator, based on a consideration of all the conditions involved.

445 William Street.

